# Clinical conditions and echocardiographic parameters associated with mortality in COVID‐19

**DOI:** 10.1111/eci.13638

**Published:** 2021-07-20

**Authors:** Angelo Silverio, Marco Di Maio, Fernando Scudiero, Vincenzo Russo, Luca Esposito, Emilio Attena, Salvatore Pezzullo, Guido Parodi, Antonello D'Andrea, Antonio Damato, Antonio Silvestro, Patrizia Iannece, Michele Bellino, Davide Di Vece, Anna Borrelli, Rodolfo Citro, Carmine Vecchione, Gennaro Galasso

**Affiliations:** ^1^ Department of Medicine, Surgery and Dentistry University of Salerno Baronissi Italy; ^2^ Division of Cardiology Eboli Hospital Salerno Italy; ^3^ Cardiology Unit Health Authority Bergamo East Bergamo Italy; ^4^ Chair of Cardiology Department of Translational Medical Sciences University of Campania “Luigi Vanvitelli” – Monaldi and Cotugno Hospital Naples Italy; ^5^ Division of Cardiology, Cardiovascular and Thoracic Department San Giovanni di Dio e Ruggi d'Aragona University Hospital Salerno Italy; ^6^ Division of Cardiology San Giuliano Hospital Naples Italy; ^7^ Division of Cardiology Villa dei Fiori Hospital Acerra Italy; ^8^ Division of Interventional Cardiology University Hospital of Sassari Sassari Italy; ^9^ Department of Cardiology and Intensive Coronary Unit “Umberto I” Hospital Nocera Inferiore Italy; ^10^ Department of Vascular Physiopathology IRCCS Neuromed Pozzilli Italy; ^11^ Department of Chemistry and Biology University of Salerno Fisciano Italy; ^12^ Department of Cardiology University Heart Center University Hospital Zurich Zurich Switzerland; ^13^ San Giovanni di Dio e Ruggi d'Aragona University Hospital Salerno Italy

**Keywords:** COVID‐19, echocardiography, left ventricular ejection fraction, outcome, SARS‐CoV‐2, tricuspid annular plane systolic excursion

## Abstract

**Background:**

Coronavirus disease 2019 (COVID‐19) is a recently recognized viral infective disease which can be complicated by acute respiratory stress syndrome (ARDS) and cardiovascular complications including severe arrhythmias, acute coronary syndromes, myocarditis and pulmonary embolism. The aim of the present study was to identify the clinical conditions and echocardiographic parameters associated with in‐hospital mortality in COVID‐19.

**Methods:**

This is a multicentre retrospective observational study including seven Italian centres. Patients hospitalized with COVID‐19 from 1 March to 22 April 2020 were included into study population. The association between baseline variables and risk of in‐hospital mortality was assessed through multivariable logistic regression and competing risk analyses.

**Results:**

Out of 1401 patients admitted at the participating centres with confirmed diagnosis of COVID‐19, 226 (16.1%) underwent transthoracic echocardiography (TTE) and were included in the present analysis. In‐hospital death occurred in 68 patients (30.1%). At multivariable analysis, left ventricular ejection fraction (LVEF, *P* < .001), tricuspid annular plane systolic excursion (TAPSE, *P* < .001) and ARDS (*P* < .001) were independently associated with in‐hospital mortality. At competing risk analysis, we found a significantly higher risk of mortality in patients with ARDS vs those without ARDS (HR: 7.66; CI: 3.95‐14.8), in patients with TAPSE ≤17 mm vs those with TAPSE >17 mm (HR: 5.08; CI: 3.15‐8.19) and in patients with LVEF ≤50% vs those with LVEF >50% (HR: 4.06; CI: 2.50‐6.59).

**Conclusions:**

TTE might be a useful tool in risk stratification of patients with COVID‐19. In particular, reduced LVEF and reduced TAPSE may help to identify patients at higher risk of death during hospitalization.

## 1 BACKGROUND

Coronavirus disease 2019 (COVID‐19) is a recently recognized infective disease caused by the severe acute respiratory syndrome coronavirus 2 (SARS‐CoV‐2), which has spread from the Hubei province, China, and has currently taken on pandemic proportions.^1^

The clinical management of patients with COVID‐19 is complex due to the need for dedicated in‐hospital pathways, protective measures for healthcare professionals and isolated beds of intensive care, particularly in areas overwhelmed by wide viral spread.^2^ Therefore, early identification of patients at higher risk for adverse outcome and prompt implementation of pharmacological and interventional treatments may be challenging.

A variety of cardiovascular complications among hospitalized patients infected by SARS‐CoV‐2, including arrhythmias, acute coronary syndromes, myocarditis and pulmonary embolism, have been reported.^3^, ^4^ Early recognition of these life‐threatening conditions is crucial for the therapeutic success.

Due to the wide availability and bedside feasibility, transthoracic echocardiography (TTE) is generally considered the first‐line imaging approach for patients with suspected or confirmed cardiac disease, particularly in critical care setting.^5^ However, the clinical context of the pandemic makes the employment of TTE difficult, since it needs close contact with the patient and may be associated with the risk of infection for echocardiographers. Therefore, it is recommended to adopt a parsimonious approach in the use of TTE, which should be carefully considered on a case‐by‐case basis and performed only if retained essential for patients care.^6^, ^7^

The role of TTE in risk stratification of patients admitted with COVID‐19 has been poorly investigated. The aim of this multicentre study was to evaluate clinical characteristics of patients hospitalized with COVID‐19 and to investigate the association between clinical characteristics and echocardiographic parameters with in‐hospital mortality.

## 2 METHODS

This is a multicentre retrospective observational study including consecutive patients with confirmed diagnosis of COVID‐19 admitted at seven Italian centres from 1 March to 22 April 2020.

COVID‐19 diagnosis was based on the World Health Organization criteria, and all cases were confirmed by real‐time reverse transcriptase‐polymerase chain reaction analysis of pharyngeal swab specimens.

All patients included in this study were evaluated by the hospital cardiology service and underwent TTE within 48 hours from admission. To minimize the exposure to COVID‐19, each referral for TTE was confirmed as clinically appropriate by one consultant cardiologist.^8^

This study was conducted according to the Declaration of Helsinki. Informed consent was waived due to the retrospective design. Reporting of the study conforms to broad EQUATOR guidelines.^9^

### 2.1 Measures and outcome

The baseline demographic, clinical, laboratory and TTE data were collected and recorded on an electronic datasheet. In all patients, demographic (age, gender, height, and weight), clinical (comorbidities, pharmacological therapy before and during hospitalization), laboratory (D‐dimer, N‐terminal pro‐brain natriuretic peptide, and high‐sensitivity troponin) and echocardiographic data were collected. TTE was performed in accordance with the current guidelines.^10^ Echocardiographic analysis included the evaluation of left ventricular end‐diastolic (LVEDV) and end‐systolic volumes (LVESV). Left ventricular systolic function was assessed by determining left ventricular ejection fraction (LVEF) through biplane analysis using the modified Simpson's rule. Systolic pulmonary artery pressure (sPAP) was derived from the tricuspid regurgitant jet velocity using systolic trans‐tricuspid pressure gradient calculated by the modified Bernoulli equation and the addition of estimated right atrial pressure according to inferior vena cava dimension and inspiratory distensibility. Heart valve regurgitations were assessed using by the Color Doppler method.^11^ As a parameter of global right ventricular (RV) function, tricuspid annular plane systolic excursion (TAPSE), which reflects the base to apex shortening of the right ventricle in systole, was assessed. After adjusting the echo transducer at the level of the RV chamber to achieve optimal visualization of the RV, TAPSE was obtained by aligning the M‐mode linear cursor to the lateral tricuspid annulus and calculated as previously described.^10^

Information on clinical course (admission in intensive care unit and respiratory support measures) and in‐hospital complications were systematically recorded. Acute respiratory distress syndrome (ARDS) diagnosis was defined according to the Berlin definition.^12^ Acute myocardial injury was diagnosed in patients with elevated cardiac troponin levels with at least one value above the 99th percentile upper reference limit.^13^

The number of patients who had died or recovered was recorded. No patient was still hospitalized at the time of the analysis (June 25, 2020). The occurrence of death during hospitalization was identified as the outcome measure of this study.

### 2.2 Statistical analysis

Distribution of continuous data was tested with the Kolmogorov‐Smirnov and the Shapiro‐Wilk test. Normally distributed variables were expressed as mean ± standard deviation, whereas non‐normal ones as median and interquartile range (IQR). Categorical variables were reported as numbers and percentages. Continuous normally distributed variables were compared by using the Student *t* test. Categorical variables were compared with chi‐squared test, or Fisher exact test when appropriate. Differences between non‐normally distributed variables were tested with Kruskal‐Wallis test. Variables from different settings (ie clinical, laboratory, echocardiographic) were tested through univariable logistic regression to evaluate the association with mortality during hospitalization. To limit the risk of overfitting, only variables significantly associated (*P* < .05) with mortality at univariable analysis were tested in the multivariable model, accounting for potential confounders and multicollinearity bias related to the interplay between them. The final regression model was built in a step‐down manner, by removing at each step the least significant predictors based on *P* values (backward elimination), reiterating this process until no nonsignificant variables remain. Variables were excluded from the regression model if showed significant collinearity, defined as a variance inflation factor >5 or a correlation coefficient >0.5. Variables describing in‐hospital treatments (eg drugs), interventions (eg respiratory support) and clinical setting (ICU, ward) were not tested in the model to avoid the introduction of unaccounted confounders related to the local protocol and patient's management in different centres. Results were presented as relative risk (RR) with their 95% confidence intervals (CI). To test the statistical interaction between ARDS and TAPSE, an interaction term ‘ARDS’ * ‘TAPSE’ was entered into a separate regression model.

A competing risk analysis for discharge free from death and in‐hospital mortality was performed and displayed using stacked cumulative incidence function area curves. The risk of in‐hospital mortality in the subgroups of interest was expressed as hazard ratio (HR) and 95% CI. LVEF >50% and TAPSE >17 normal cut‐off values were considered for subgroup definitions.^10^, ^14^

For all test, a *P* value < .05 was considered statistically significant. Analyses were performed by using R version 3.5.1 (R Foundation for Statistical Computing, Vienna, Austria).

## 3 RESULTS

A total of 1401 patients with confirmed diagnosis of COVID‐19 were admitted at the participating centres; in‐hospital mortality was reported in 181 cases (12.9%; CI: 11.1%‐14.8%).

Out of the entire population, 226 (16.1%) subjects underwent TTE within 48 hours from admission and were included in this analysis. In‐hospital death occurred in 68 cases (30.1%; CI: 24.2%‐36.5%); 158 patients recovered and were discharged. The baseline demographic and clinical features of the study population at admission are summarized in Table 1. The mean age was 68.9 ± 13.9 years; male sex was reported in 141 patients (62.4%) and was significantly prevalent among patients who died during hospitalization as compared with those who recovered (75.0% vs 57.0%, *P* = .016). At admission, the most common symptoms were dyspnoea (157, 69.5%), fever (153, 67.7%), and cough (87, 38.5%). Fatigue was reported in 77 patients (34.1%) and was prevalent in patients who underwent fatal outcome (47.1% vs 28.5%, *P* = .011).

**TABLE 1:** Demographic and clinical features at admission

	Overall (N = 226)	Death (N = 68)	Survival (N = 158)	*P*
Age, years	68.9 ± 13.9	72.4 ± 12.1	67.4 ± 14.3	.012
Males, N (%)	141 (62.4)	51 (75.0)	90 (57.0)	.016
Height, cm	165.2 ± 8.3	165.9 ± 8.0	164.8 ± 8.4	.508
Weight, kg	74.3 ± 13.1	74.4 ± 16.1	74.2 ± 11.6	.946
Clinical presentation				
Symptoms onset to hospitalization, d (median [IQR])	5.50 [2.25, 10.00]	5.00 [2.00, 10.00]	6.00 [3.00, 10.00]	.244
Dyspnoea, N (%)	157 (69.5)	53 (77.9)	104 (65.8)	.098
Chest tightness, N (%)	69 (30.5)	27 (39.7)	42 (26.6)	.071
Fever, N (%)	153 (67.7)	52 (76.5)	101 (63.9)	.090
Cough, N (%)	87 (38.5)	26 (38.2)	61 (38.6)	1.000
GI symptoms, N (%)	30 (13.3)	9 (13.2)	21 (13.3)	1.000
Syncope, N (%)	21 (9.3)	5 (7.4)	16 (10.1)	.683
Medical history				
Hypertension, N (%)	138 (61.1)	48 (70.6)	90 (57.0)	.075
Diabetes, N (%)	64 (28.3)	23 (33.8)	41 (25.9)	.296
Dyslipidaemia, N (%)	62 (30.8)	18 (29.5)	44 (31.4)	.916
Smoking, N (%)	42 (18.6)	14 (20.6)	28 (17.7)	.748
CAD, N (%)	37 (16.4)	15 (22.1)	22 (13.9)	.187
Prior MI, N (%)	35 (15.5)	14 (20.6)	21 (13.3)	.234
Prior PCI, N (%)	36 (15.9)	14 (20.6)	22 (13.9)	.290
Prior CABG, N (%)	13 (5.8)	8 (11.8)	5 (3.2)	.025
HF, N (%)	22 (9.7)	11 (16.2)	11 (7.0)	.058
History of AF, N (%)	46 (20.4)	14 (20.6)	32 (20.4)	1.000
History of VT, N (%)	3 (1.3)	2 (2.9)	1 (0.6)	.449
History of SVT, N (%)	7 (3.1)	1 (1.5)	6 (3.8)	.612
Prior stroke/TIA, N (%)	18 (8.0)	5 (7.4)	13 (8.2)	1.000
COPD, N (%)	46 (20.4)	17 (25.0)	29 (18.4)	.338
CKD, N (%)	45 (19.9)	19 (27.9)	26 (16.5)	.072
Malignancy, N (%)	27 (11.9)	10 (14.7)	17 (10.8)	.538
Home pharmacotherapy				
ACE inhibitors, N (%)	61 (27.0)	24 (35.3)	37 (23.4)	.093
ARBs, N (%)	38 (16.8)	10 (14.7)	28 (17.7)	.717
ASA, N (%)	67 (29.6)	27 (39.7)	40 (25.3)	.044
P2Y12 inhibitors, N (%)	21 (9.3)	8 (11.8)	13 (8.2)	.555
VKA, N (%)	9 (4.0)	4 (5.9)	5 (3.2)	.557
NOACs, N (%)	33 (14.6)	9 (13.2)	24 (15.2)	.860
Diuretics, N (%)	47 (20.8)	20 (29.4)	27 (17.1)	.056
Statins, N (%)	71 (31.4)	25 (36.8)	46 (29.1)	.327
Insulin, N (%)	32 (14.2)	12 (17.6)	20 (12.7)	.436

Note

Continuous normally distributed variables are expressed as mean ± SD. Categorical variables are n (%). Continuous non‐normally distributed variables are median (interquartile range).

Abbreviations: ACE, angiotensin‐converting enzyme; AF, atrial fibrillation; ARBs, angiotensin II receptor blockers; ASA, acetylsalicylic acid; CABG, coronary artery bypass graft; CAD, coronary artery disease; CKD, chronic kidney disease; COPD, chronic obstructive pulmonary disease; GI, gastrointestinal; HF, heart failure; IQR, interquartile range; MI, myocardial infarction; NOACs, nonvitamin‐K‐dependent oral anti‐coagulants; PCI, percutaneous coronary intervention; SVT, supraventricular tachycardia; TIA, transient ischaemic attack; VKA, vitamin K antagonists; VT, ventricular tachycardia.

Hypertension (138, 61.1%), dyslipidaemia (62, 30.8%) and diabetes (64, 28.3%) were the most common comorbidities, with no statistical difference between patients who died vs those who recovered. History of coronary artery disease, prior myocardial infarction and heart failure (HF) had higher prevalence in patients deceased during hospitalization, albeit not reaching statistical significance. Thirty‐six patients underwent prior percutaneous coronary intervention and 13 coronary artery bypass graft, which was significantly prevalent in the deceased cohort (11.8% vs 3.2%, *P* = .025).

At admission, 99 patients (43.8%) were being treated with renin‐angiotensin‐aldosterone system (RAAS) inhibitors with no difference between deceased vs survived patients; oral anticoagulation therapy was reported in 42 cases (18.6%).

Main clinical, laboratory and echocardiographic characteristics of the study population are summarized in Table 2. Admission in intensive care unit was required for 72 patients (31.9%) and was higher in deceased patients as compared with those who recovered (67.6% vs 16.5%, *P* < .001). The incidence of ARDS (83.8% vs 31.0%, *P* < .001) as well as the need of oxygen therapy, noninvasive and invasive ventilatory support was higher in patients who underwent fatal outcome during the hospitalization. The rate of cardiovascular in‐hospital complications, including myocardial injury (54.4% vs 20.3%; *P* < .001), myocardial infarction (20.6% vs 6.3%; *P* = .003), pulmonary embolism (23.5% vs 10.1%; *P* = .015) and acute HF (36.8% vs 8.9%; *P* < .001) was significantly higher among deceased patients as compared with those who recovered. None of the patients included in this study underwent coronary angiography during the hospital stay.

**TABLE 2:** Clinical, laboratory and echocardiographic findings during hospitalization

	Overall (N = 226)	Death (N = 68)	Survival (N = 158)	*P*
Laboratory				
Peak troponin, ng/L (median [IQR])	24.40 [2.78, 225.00]	98.00 [24.40, 400.00]	20.40 [2.76, 123.38]	.004
Peak D‐dimer, ng/mL (median [IQR])	625.00 [100.75, 1994.00]	1616.00 [568.00, 2735.00]	524.50 [59.50, 1296.25]	.009
NT‐proBNP, pg/mL (median [IQR])	1100.00 [300.00, 3442.00]	2417.50 [502.50, 7010.00]	731.00 [200.00, 2972.00]	.098
Echocardiography				
LVEF, %	53.1 ± 8.7	47.6 ± 8.8	55.4 ± 7.6	<.001
LVEF ≤50%, N (%)	53 (23.5)	33 (48.5)	20 (12.7)	<.001
LVEDV, mL	105.7 ± 31.8	110.9 ± 36.4	103.4 ± 29.5	.111
LVESV, mL	50.9 ± 20.7	59.2 ± 28.1	47.2 ± 15.1	<.001
TAPSE, mm	20.5 ± 4.3	17.5 ± 4.2	21.7 ± 3.6	<.001
TAPSE ≤17 mm, N (%)	47 (20.8)	32 (47.1)	15 (9.5)	<.001
sPAP, mm Hg	35.5 ± 9.7	40.9 ± 10.3	33.3 ± 8.4	<.001
Pericardial effusion, N (%)	13 (13.1)	5 (17.2)	8 (11.4)	.651
Moderate‐severe MR, N (%)	36 (15.9)	15 (22.1)	21 (13.3)	.146
Moderate‐severe TR, N (%)	48 (21.2)	26 (38.2)	22 (13.9)	<.001
Moderate‐severe AR, N (%)	4 (1.8)	0 (0.0)	4 (2.5)	.439
Moderate‐severe AS, N (%)	7 (3.1)	3 (4.4)	4 (2.5)	.742
In‐hospital course				
LMWH, N (%)	183 (81.7)	59 (88.1)	124 (79.0)	.156
Antiviral drugs, N (%)	118 (52.2)	46 (67.6)	72 (45.6)	.004
Glucocorticoids, N (%)	102 (45.1)	39 (57.4)	63 (39.9)	.023
Immunoglobulins, N (%)	2 (0.9)	0 (0.0)	2 (1.3)	.875
Antibiotics, N (%)	166 (73.5)	59 (86.8)	107 (67.7)	.005
Tocilizumab, N (%)	1 (1.0)	1 (3.4)	0 (0.0)	.636
Hydroxychloroquine, N (%)	180 (79.6)	59 (86.8)	121 (76.6)	.118
ICU, N (%)	72 (31.9)	46 (67.6)	26 (16.5)	<.001
Oxygen therapy, N (%)	190 (84.1)	65 (95.6)	125 (79.1)	.004
Noninvasive ventilation, N (%)	99 (43.8)	49 (72.1)	50 (31.6)	<.001
Invasive ventilation, N (%)	67 (29.6)	45 (66.2)	22 (13.9)	<.001
ARDS, N (%)	106 (46.9)	57 (83.8)	49 (31.0)	<.001
Pulmonary embolism, N (%)	32 (14.2)	16 (23.5)	16 (10.1)	.015
Myocardial injury, N (%)	69 (30.5)	37 (54.4)	32 (20.3)	<.001
MI, N (%)	24 (10.6)	14 (20.6)	10 (6.3)	.003
Acute HF, N (%)	39 (17.3)	25 (36.8)	14 (8.9)	<.001
Death, N (%)	68 (30.1)	68 (100.0)	0 (0.0)	<.001

Note

Continuous normally distributed variables are expressed as mean ± SD. Categorical variables are n (%). Continuous non‐normally distributed variables are median (interquartile range).

Abbreviations: AR, aortic regurgitation; ARDS, acute respiratory distress syndrome; AS, aortic stenosis;BNP, brain natriuretic peptide; HF, heart failure; ICU, intensive care unit; IQR, interquartile range; LMWH, low‐molecular‐weight heparin; LVEDV, left ventricular end‐diastolic volume; LVEF, left ventricular ejection fraction; LVESV, left ventricular end‐systolic volume; MI, myocardial infarction; MR, mitral regurgitation; NT‐proBNP, N‐terminal pro‐B‐type natriuretic peptide; sPAP, systolic pulmonary artery pressure; TAPSE, tricuspid annular plane excursion; TR, tricuspid regurgitation.

Patients who experienced in‐hospital mortality had lower LVEF (47.6 ± 8.8% vs 55.5 ± 7.6%; *P* < .001) and TAPSE (17.5 ± 4.2 mm vs 21.7 ± 3.6 mm; *P* < .001) and higher LVESV (59.2 ± 28.1 vs 47.2 ± 15.1 mL, *P* < .001) as compared with patients who survived. The proportion of moderate‐to‐severe tricuspid regurgitation (TR, 38.2% vs 13.9%, *P* < .001) and sPAP values (40.9 ± 10.3 vs 33.3 ± 8.4; *P* < .001) were higher in the deceased cohort.

The following covariates were entered into the multivariable logistic regression model: LVEF, TAPSE, sPAP, male sex, age, prior coronary artery bypass graft, moderate‐to‐severe TR, and ARDS. After adjustment for confounders, reduced LVEF (RR: 0.93; CI: 0.89‐0.97; *P* < .001), reduced TAPSE (RR: 0.80; CI: 0.72‐0.88; *P* < .001) and ARDS (RR: 3.05; CI: 2.69‐3.23; *P* < .001) resulted to be independently associated with in‐hospital mortality (Table 3). There was no statistically significant interaction between ARDS and TAPSE.

**TABLE 3:** Univariable and multivariable analysis for the risk of in‐hospital mortality

	Univariable			Multivariable		
	RR	CI	*P*	RR	CI	*P*
Age	1.02	1‐1.04	.013	‐	‐	NS
Male sex	1.81	1.17‐2.61	.011	‐	‐	NS
Prior CABG	2.18	1.2‐3	.017	‐	‐	NS
LVEF	0.92	0.89‐0.95	<.001	0.93	0.89‐0.97	<.001
LVESV	1.02	1.01‐1.03	<.001	‐	‐	NS
TAPSE	0.81	0.75‐0.87	<.001	0.80	0.72‐0.88	<.001
sPAP	1.06	1.04‐1.09	<.001	‐	‐	NS
Moderate‐severe TR	2.3	1.61‐2.96	<.001	‐	‐	NS
ARDS	5.87	4.01‐7.81	<.001	3.95	2.69 ‐ 3.23	<.001
Pulmonary embolism	1.87	1.18‐2.55	.010	‐	‐	NS
Myocardial injury	2.39	1.96‐3.46	<.001	‐	‐	NS
Acute HF	2.39	1.85‐2.81	<.001	‐	‐	NS

Abbreviations: ARDS, acute respiratory distress syndrome; CABG, coronary artery bypass graft; CI, confidence interval; HF, heart failure; LVEF, left ventricular ejection fraction; RR, relative risk; sPAP, systolic pulmonary artery pressure; TAPSE, tricuspid annular plane excursion; TR, tricuspid regurgitation.

**FIGURE 1:**
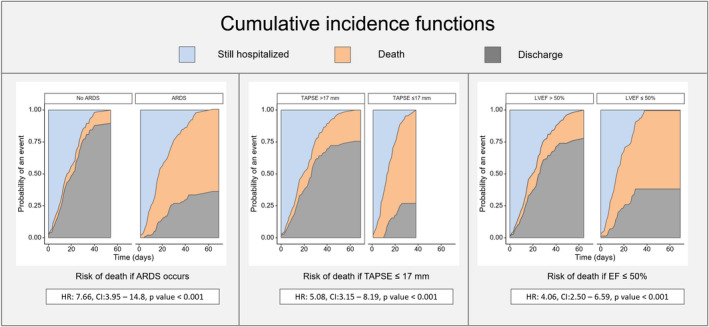
Stacked cumulative incidence function area curves for discharge free from death and in‐hospital mortality according to ARDS, LVEF and TAPSE values. The probability of discharge free from death over the course of the study is shown in blue. The probability of in‐hospital mortality is shown as red. The area in grey is the probability of being event free. ARDS, acute respiratory distress syndrome; CI, confidence interval; HR, hazard ratio; LVEF, left ventricular ejection fraction; TAPSE, tricuspid annular plane excursion

The risk of in‐hospital death according to the presence of ARDS, low LVEF and low TAPSE values was estimated considering discharge alive as competing risk. Patients with ARDS showed a significantly higher risk of in‐hospital death than those without ARDS (HR: 7.66; CI: 3.95‐14.8; *P* < .001; Figure 1). By stratifying the study population according to the LVEF and TAPSE cut‐off normal values,^10^, ^14^ we found a significantly higher mortality risk in patients with TAPSE ≤17 mm vs those with TAPSE >17 mm (HR: 5.08; CI: 3.15‐8.19; *P* < .001) and in patients with LVEF ≤50% vs those with LVEF >50% (HR: 4.06; CI: 2.50‐6.59; *P* < .001; Figure 1).

## 4 DISCUSSION

The main findings of the present multicentre Italian study can be summarized as follows: (a) echocardiography was performed in about one sixth of patients hospitalized for COVID‐19; (b) the in‐hospital course of this COVID‐19 population was characterized by a high need for intensive treatments and a high incidence of severe adverse events; (c) LVEF, TAPSE and ARDS were independently associated with in‐hospital mortality; (d) at competing risk analysis, patients with ARDS, TAPSE ≤17 mm ad LVEF ≤50% emerged as the highest risk groups for death during hospitalization.

Italy has been one of the hardest‐hit Country by COVID‐19, and the Italian government has adopted an early nationwide community containment to control spreading of infection.^15^, ^16^ In this observational registry, we recruited 1401 COVID‐19 patients hospitalized at seven Italian centres; of them, 16.1% underwent TTE. This proportion of use of ultrasound may be considered consistent with the recommendations proposed by the leading societies of cardiovascular imaging intended to optimize sources and reduce the risk of contagion in this clinical setting.^6^, ^7^ The strict indication for TTE may contribute to explain the high proportion of comorbidities and in‐hospital complications observed in this study population. In fact, our patients showed a worse clinical profile, more need of intensive care, and a higher incidence of in‐hospital death, compared to previous study cohorts.^17^, ^18^

Many authors reported a high risk of multiple and life‐threatening cardiovascular complications during the in‐hospital course of patients infected by SARS‐CoV‐2, including pulmonary embolism, myocardial infarction, acute myocardial injury, acute HF and ventricular arrhythmias.^19^, ^20^, ^21^, ^22^, ^23^ These cases need to be prioritized for focused cardiac ultrasound studies, which have been proposed as the most appropriate approach for minimizing the time of exposure with the patients and improve the safety of operators.^6^ Therefore, we collected conventional meaningful parameters of LV and RV function, valves and pericardium. Data on advanced TTE, such as speckle‐tracking echocardiography, were not systematically collected, probably because they are not easy to acquire routinely in patients infected by SARS‐CoV‐2 (need for multiple views, ECG gating, adequate frame rate, multiple cardiac cycles cine loops).

At multivariable analysis, LVEF, TAPSE and ARDS emerged as independent predictors of in‐hospital mortality. Our results were consistent with previous findings from Rath and colleagues, who reported that impaired LVEF and RV systolic function were associated with higher mortality in 123 patients with COVID‐19.^24^

Left ventricular ejection fraction is a well‐established parameter for risk stratification both in the ward and in the intensive care unit, and still represent the most widely used parameter for the assessment of LV systolic function.^10^, ^25^, ^26^ In patient with reduced LVEF, COVID‐19 could act like a precipitating factor and rapidly deteriorate the patients' clinical status. In fact, previous studies demonstrated the association of concomitant cardiac disease with outcome in patients infected by SARS‐CoV‐2.^27^

Although SARS‐CoV‐2 primarily affects the respiratory tract, it has also been associated with LV dysfunction in more serious cases.^28^ The pathophysiology of myocardial impairment has not yet definitely clarified, but either ischaemic or nonischaemic mechanisms have been hypothesized, including systemic inflammatory response, hypoxia‐induced cardiac injury, direct viral damage and adrenergic stimulation.^29^, ^30^ Indeed, the occurrence of myocardial damage demonstrated by increased cardiac troponin levels have been associated with in‐hospital mortality in patients with COVID‐19.^19^ Moreover, in a Chinese cohort from Wuhan, acute HF and myocardial injury were significantly associated with death regardless of past medical history of cardiovascular diseases.^31^

Independently from the time of onset of LV dysfunction, this analysis suggests that LVEF at admission may be very useful to identify patients with higher probability of fatal outcome, and the reference normal value of 50% might be effectively employed also in this patient population.

An extensive pulmonary interstitial involvement, thrombotic complications such as pulmonary embolism, and the use of high positive end‐expiratory pressures, are some of the mechanisms potentially involved in a RV impairment in patients with COVID‐19.^32^, ^33^ In a cohort of 74 patients infected by SARS‐CoV‐2 assessed by TTE, the most common abnormalities were RV dilation and dysfunction.^34^ Moreover, Pagnesi et al^35^ showed that patients with RV dysfunction had a higher burden of cardiac pre‐existing comorbidities. In the present study, we found that the RV longitudinal shortening estimated by TAPSE was an independent predictor of mortality, and abnormal TAPSE values identified individuals with about five times greater risk of death during the hospitalization. According to the pathophysiology of the disease, RV function is essential to maintain a clinical balance and its evaluation during the progression of the infection may be crucial for the patient risk assessment.^24^, ^36^ This information could drastically influence their clinical management, especially in terms of diuretic therapy, ventilation strategies and specific anticoagulation regimens. A quick and easy parameter such as TAPSE is suitable for assessing RV function in these patients, especially for its rapid and real‐time feasibility in urgent and intensive care settings. Our results are also consistent with a recent multicentre study including 870 COVID‐19 patients undergoing TTE during the acute phase, which identified LV longitudinal strain and RV free wall strain, two indices of left and right ventricular systolic function, respectively, as independently associated with 1‐year mortality.^37^

In this study population, we found a very high prevalence of ARDS. Consistently with previous data from Chinese cohorts, the prevalence of cardiovascular diseases, in particular hypertension, was significantly increased in critically ill patients with ARDS compared to those with milder forms.^38^ The high proportion of ARDS might have contributed to the mortality rate observed, as the association between ARDS and fatal outcome in patients hospitalized for COVID‐19 has been extensively described.^39^, ^40^

Although limited by the retrospective nature of the study, our results point out the usefulness of TTE for risk stratification in COVID‐19 patients. An accurate evaluation of both LV and RV function by echocardiography should be strongly considered in this setting, particularly in more critical and severe cases.^41^

Some limitations of this observational retrospective study should be acknowledged. Although we reviewed all consecutive patients infected by SARS‐CoV‐2 hospitalized at different Italian institutions, TTE was performed in only one sixth of the entire patient population. The need of echocardiographic data restricted the analysis to 226 cases, who could not be representative of the entire COVID‐19 population and may have affected the generalizability of our findings. The use of TTE in only a limited percentage of cases, which resulted to be particularly serious and at high risk of adverse events, may reflect the adherence to the current recommendation on the use of imaging modalities in patients with COVID‐19, which circumscribe the adoption of TTE to more challenging cases.^6^, ^8^ Indeed, the high mortality rate observed in our study was consistent with previous echocardiography‐focused studies, which reported similarly high rates of mortality during the in‐hospital stay.^34^, ^37^

In this study, we did not include indexes of diastolic dysfunction as well as novel parameters from speckle tracking or other advanced echocardiographic techniques. The context of pandemic and the risk of infection for the operators make difficult to perform a lot of measures and may justify an approach based on few parameters quick to assess at bedside.

Owing to the absence of TTE data before hospitalization, we cannot exclude the presence of patients with pre‐existent LV and/or RV impairment in this data set. However, our aim was not to investigate the prognostic role of new‐onset TTE abnormalities, but to explore the association between the presence of echocardiographic abnormalities at admission and in‐hospital course in patients with COVID‐19. Therefore, the analysis was restricted to patients who underwent TTE within 48 hours from admission.

## 5 CONCLUSIONS

Cardiovascular complications can negatively impact on outcomes of patients with COVID‐19. Clinical and echocardiographic parameters such as LVEF ≤50%, TAPSE ≤17 mm, and ARDS might help to identify patients at higher risk for in‐hospital mortality. Our preliminary findings need to be confirmed in larger, prospective studies.

## CONFLICT OF INTEREST

No conflict of interest.
